# Environmental Application of a Bacteriophage Cocktail Reduces Antibiotic-Resistant *Escherichia coli* in Poultry Litter Without Disrupting Gut Microbiota

**DOI:** 10.3390/ani15172525

**Published:** 2025-08-27

**Authors:** Marta Kuźmińska-Bajor, Maciej Kuczkowski, Damian Konkol, Mariusz Korczyński, Magdalena Rakicka-Pustułka, Sylwia Kozioł, Ludwika Tomaszewska-Hetman, Anita Rywińska

**Affiliations:** 1 Department of Biotechnology and Food Microbiology, Wroclaw University of Environmental and Life Sciences, 37 Chełmońskiego St., 51-630 Wroclaw, Poland; magdalena.rakicka-pustulka@upwr.edu.pl (M.R.-P.); ludwika.tomaszewska-hetman@upwr.edu.pl (L.T.-H.); anita.rywinska@upwr.edu.pl (A.R.); 2 Department of Epizootiology and Clinic of Birds and Exotic Animals, Wroclaw University of Environmental and Life Sciences, 45 Grunwaldzki Sq., 50-366 Wroclaw, Poland; maciej.kuczkowski@upwr.edu.pl; 3 Department of Animal Nutrition and Feed Sciences, Wroclaw University of Environmental and Life Sciences, 38D Chełmońskiego St., 51-630 Wroclaw, Poland; damian.konkol@upwr.edu.pl (D.K.); mariusz.korczynski@upwr.edu.pl (M.K.); 4 Department of Biophysics, Faculty of Biotechnology, University of Wroclaw, Joliot-Curie 14a, 50-387 Wroclaw, Poland

**Keywords:** *E. coli*, AMR, bacteriophages, phage treatment, broiler chickens, litter

## Abstract

The increasing demand for poultry meat calls for sustainable production methods that address animal welfare and combat antimicrobial resistance (AMR). Commensal *Escherichia coli* serve as reservoirs of resistance genes that may transfer to pathogens, facilitating AMR spread in agriculture. This study evaluated the efficacy of a bacteriophage cocktail, UPWr_E, applied as a litter spray to reduce total and antibiotic-resistant *E. coli* in broiler chicken rearing. The cocktail, containing four lytically active phages, was administered for four weeks. Microbiological analyses of litter, feces, and cecal contents showed a significant reduction in total *E. coli* by 3.2 log_10_ CFU/g in litter and a decrease in resistant strains to gentamicin, enrofloxacin, tetracycline, and sulfamethoxazole–trimethoprim, compared to controls. No significant changes occurred in *E. coli* loads in feces or cecal contents, indicating limited impact on the number of commensal *E. coli* in cecal contents. Phages remained detectable and stable in litter and feces throughout the study. These findings demonstrate the potential of phage therapy as a targeted, environmentally friendly approach to control AMR reservoirs in poultry farming. Incorporating phage-based treatments into AMR management strategies could improve food safety and promote sustainable animal production.

## Simple Summary

The spread of antimicrobial-resistant (AMR) *Escherichia coli* strains in poultry farming is a significant challenge for animal health, food safety, and public health. Traditional antibiotic treatments are becoming less effective due to increasing resistance, highlighting the urgent need for alternative control strategies. Bacteriophages are viruses that specifically infect and kill bacteria and represent a promising tool to combat resistant bacteria without harming beneficial microorganisms or contributing to resistance development. In this study, four bacteriophages, UPWr_E1, UPWr_E2, UPWr_E3, and UPWr_E4, previously characterized for their activity against avian pathogenic *E. coli* (APEC), were tested for their efficacy in reducing drug-resistant *E. coli* populations in poultry litter. The results demonstrated that phage treatment significantly decreased the number of resistant bacteria in the litter environment, which is a critical reservoir for the spread of AMR in poultry farms. By reducing bacterial loads in the litter, phage application may improve farm hygiene and decrease the risk of resistant bacteria transmission between animals and to humans. These findings support the potential of phage therapy as a complementary or alternative approach to antibiotics in poultry production, contributing to sustainable farming practices and helping to mitigate the global AMR crisis.

## 1. Introduction

A major challenge associated with the rapidly increasing global population is the sustainable production of sufficient animal-derived protein to meet escalating nutritional demands [1]. Poultry meat is the most consumed meat worldwide, and its consumption has risen substantially over the past decades [2]. In response, the industry has established large-scale poultry production systems capable of meeting this demand. However, this intensive and industrial-scale broiler production facilitates both horizontal and vertical transmission of pathogens within flocks.

Among the most prevalent bacterial pathogens in broiler chickens are *Escherichia coli*, *Clostridium perfringens*, and *Enterococcus cecorum*. Colibacillosis, caused by avian pathogenic *E. coli*, is a major disease complex in broiler chickens worldwide, manifesting as both localized and systemic infections [3]. Importantly, *E. coli* acts not only as an opportunistic pathogen but also as a commensal organism within the intestinal microbiota of both animals and humans [4].

According to the European Food Safety Authority (EFSA) report on Animal Health and Welfare (2021) [4], *E. coli* strains isolated from poultry exhibit resistance to multiple antimicrobial agents, including ampicillin/amoxicillin, colistin, polymyxin B, enrofloxacin/ciprofloxacin, gentamicin, neomycin, spectinomycin, streptomycin, sulfamethoxazole–trimethoprim, and several tetracyclines such as oxytetracycline, doxycycline, and chlortetracycline. As part of the European One Health Action Plan against Antimicrobial Resistance (AMR), the European Commission has committed to revising EU legislation on the harmonized monitoring of AMR in zoonotic and commensal bacteria from food-producing animals and food [5].

Several studies have shown that monitoring AMR in the commensal indicator *E. coli* is an effective approach for identifying emerging AMR threats in food-producing animals, with significant implications for public health [6,7]. Beyond its public health relevance, AMR surveillance in commensal *E. coli* is also valuable for veterinary medicine. Veterinary prescription guidelines are informed by AMR trends observed in both commensal and clinical isolates of *E. coli* [8]. Although not directly linked to clinical disease, commensal *E. coli* are recognized as reservoirs of resistance genes, which can be transferred to pathogenic bacteria.

Antibiotic treatment promotes resistance in commensal *E. coli* through natural selection following antimicrobial exposure [9]. Notably, acquired resistance can be horizontally transferred to pathogenic bacteria via mobile genetic elements, such as plasmids and transposons [10]. Human exposure to viable, antibiotic-resistant *E. coli* may occur via contaminated surfaces, interaction with polluted environments, direct contact with livestock, or consumption of undercooked poultry products [11].

Furthermore, poultry litter, which is frequently applied as organic fertilizer, may harbor resistant bacteria and antimicrobial resistance genes. As such, it could serve as a potential vector for AMR dissemination from poultry to humans through the food chain [12,13].

In light of these challenges, there is an urgent need to explore effective strategies to mitigate AMR in poultry litter. Among these, bacteriophages have gained attention as a promising biocontrol tool, owing to their ability to specifically target and eliminate bacterial populations [14,15]. The administration of bacteriophages to poultry litter has shown efficacy in modulating the microbial composition and diversity, while reducing the prevalence of undesirable and pathogenic bacteria [13,15].

Integrating phage-based interventions into poultry litter management aligns with the One Health framework, supporting improved animal welfare, reduced antimicrobial use, and enhanced public health outcomes. Given the significant potential of bacteriophages as anti-AMR agents, this study was conducted to evaluate the efficacy of the phage cocktail UPWr_E, applied as a litter spray in an experimental poultry house, in reducing the population of drug-resistant *E. coli*.

## 2. Materials and Methods

### 2.1. Bacteriophages

The bacteriophages UPWr_E1, UPWr_E2, UPWr_E3, and UPWr_E4 were applied in this study. They were previously isolated from urban wastewater samples collected at the Wrocław treatment plant, as described by Śliwka et al. (2025) [16]. Taxonomic classification based on genomic analysis revealed that UPWr_E1 belongs to the genus *Krischvirus* within the family *Straboviridae*, while both UPWr_E2 and UPWr_E4 are members of the genus *Tequatrovirus* within the subfamily *Tevenvirinae*. UPWr_E3 belongs to the genus *Phapecoctavirus*, being a part of the *Stephanstirmvirinae* family. In vitro lytic activity assays demonstrated that UPWr_E1 and UPWr_E4 exhibited a lysis efficacy of 64% against a panel of 142 APEC strains. UPWr_E3 was able to infect 58% of APEC strains, whereas UPWr_E2 displayed a lysis rate of 46% [16]. Comprehensive in silico genome analyses of the abovementioned bacteriophages confirmed the absence of known virulence factors, toxins, or genes associated with pathogenicity in *E. coli* or other bacterial pathogens. Given their notable anti-APEC activity and favorable genomic safety profile [16], these phages have been proven in vivo to be effective in reducing the number of APEC in murine and chicken experimental models [17]. To further investigate their potential to mitigate undesirable bacteria in poultry production, they were subsequently tested for their ability to decrease the number of resistant *E. coli* in poultry litter.

### 2.2. Bacteriophage Propagation and Cocktail Preparation

The amplification of UPWr_E1, UPWr_E2, and UPWr_E4 phages was performed on the APEC 158B host strain, while amplification of UPWr_E3 was performed on APEC 258. Phages were amplified using a method described elsewhere [16,17]. Briefly, bacterial cultures were inoculated on 10 mL of LB broth with a single colony of the appropriate APEC strain, followed by overnight incubation at 37 °C with shaking at 150 rpm. After incubation, 0.5 mL of the overnight culture was inoculated into 10 mL of LB broth and incubated until the optical density (OD600 nm) reached 0.2. In the next step, the bacterial culture was centrifuged for 10 min at 5000× *g* to remove any remaining cell debris and filtered through 0.22 µm pore size syringe filters. Then, 5 mL of the resulting phage lysate, from the first step of propagation, was added to 150 mL of the host culture (OD600 nm = 0.2) inoculated with 200 µL of the overnight culture, and incubated overnight at 37 °C. As a last step, the centrifugation and filtration steps were repeated. Bacteriophage titer was determined using the double-layer agar method described by Adams (1959) [18]. As a phage mixture, the cocktail UPWr_E containing phages UPWr_E1, UPWr_E2, UPWr_E3 and UPWr_E4 was formulated from sterile phage preparations, which were diluted in PBS and mixed in an equal ratio to obtain a final titer of between 10^8^–10^9^ PFU/mL for each phage.

### 2.3. Experimental Design and Animal Housing

In vivo experiments were conducted using 14-day-old Ross 308 male and female broiler chickens, obtained from a local farm and placed on deep litter in premises meeting the required zootechnical conditions. Only healthy birds exhibiting normal appearance and behavior were included in the analysis. A total of 240 birds were randomly assigned to two experimental groups (120 per group). Each group was housed separately under controlled environmental conditions at an ambient temperature of 25 °C. Within each group, 20 birds were distributed across six wire pens (*n* = 6) to ensure balanced distribution for statistical analysis. Each pen was equipped with one feeder (stainless steel, 20 kg capacity, 41 cm diameter) and one drinker (plastic, 10 L capacity, 38 cm diameter) and was bedded with wheat straw. Pen dimensions (1 m × 1.2 m) were selected to achieve a stocking density of 36 kg/m^2^ by day 42 of the birds’ life. Birds were provided with commercial broiler feed (Broiler Grower II, Tasomix, Poland) and water ad libitum throughout the experiment. The experimental period lasted 28 days. All procedures involving animals were reviewed and approved by the Animal Welfare Committee of the Faculty of Veterinary Medicine, Wrocław University of Environmental and Life Sciences (protocol code 1K.2022).

### 2.4. Treatment and Sampling Protocol

To evaluate the efficacy of the UPWr_E bacteriophage cocktail in reducing resistant bacteria in litter, the following experimental treatments were applied: group 1 served as the control and received 300 mL of sterile phosphate-buffered saline (PBS) sprayed onto the litter on days 0, 7, 14 and 21 of the experimental period. Group 2 received the UPWr_E bacteriophage cocktail, applied in PBS at a concentration of 10^8^–10^9^ PFU/mL, on the same days. Prior to each litter treatment, samples were collected for microbiological analysis. Both sampling and subsequent litter treatments were conducted sequentially, with each pen handled one at a time, in order to minimize the number of additional interventions. For each pen, 5 litter samples were collected from at least five distinct locations, approximately 20–30 cm from each of the four corners and from the center of the pen. Equal litter subsamples, each weighing approximately 4 g, were collected from each pen and combined to form a total sample of 20 g. Litter and fresh fecal samples were collected simultaneously in the morning from each pen. Between 5 and 10 of the freshest droppings were randomly selected to obtain a total fecal sample of 20 g. All samples were separately homogenized with 80 mL of PBS using a stomacher (BagMixer 400P, Interscience, Saint-Nom-la-Bretèche, France) for 1 min. Serial tenfold dilutions in sterile PBS were prepared, and 100 µL aliquots were plated onto MacConkey agar to quantify total presumptive *Escherichia coli* counts and MacConkey agar plates supplemented with the respective antibiotics. Selective plating was performed on MacConkey agar supplemented with antibiotics, including 1.0 mg/L cefotaxime representing third generation cephalosporins, 2.0 mg/L colistin representing polymyxins, 2.0 mg/L gentamicin representing aminoglycosides, 0.125 mg/L enrofloxacin representing fluoroquinolones, 8 mg/mL tetracycline representing tetracyclines, and 0.5 mg/mL sulfamethoxazole with trimethoprim representing sulfonamides, to assess the prevalence of antibiotic-resistant presumptive *E. coli* strains. Antibiotics were added from concentrated stock solutions. They were diluted into the medium after autoclaving and cooling to approximately 50 °C to obtain the appropriate final concentrations and ensure uniform distribution in the plates. Non-selective detection of *Escherichia coli* was performed following European Committee on Antimicrobial Susceptibility Testing (EUCAST) and Clinical and Laboratory Standards Institute (CLSI) guidelines to ensure standardized isolation and identification procedures [19]. Presumptive *E. coli* organisms were considered lactose-fermenting colonies with size and morphology consistent with those of *E. coli* among other *Enterobacteriaceae*.

### 2.5. Cecal Content Analysis

After the experiment (day 28 of study; day 42 of bird life), three birds per pen (18 birds per group) were randomly selected and euthanized for post-mortem examination. Cecal content samples were collected aseptically for microbiological analysis. Quantification of *Enterobacteriaceae* populations was performed by plating on MacConkey and MacConkey agar supplemented with 1.0 mg/L cefotaxime, 2.0 mg/L colistin, 2.0 mg/L gentamicin, 0.125 mg/L enrofloxacin, 8 mg/mL tetracycline, and 0.5 mg/mL sulfamethoxazole combined with trimethoprim. The presence and concentration of bacteriophages in cecal contents were determined using the double-layer agar method on both host strains APEC 158B and APEC 258. Bacterial and phage loads were expressed as colony-forming units (CFUs) and plaque-forming units (PFUs) per gram of cecal content, respectively.

### 2.6. Productive Performance

On day 28 of the experiment, individual body weights of the birds were recorded. Throughout the experimental period, daily measurements of feed intake, feed conversion ratio (FCR), and mortality rate were collected.

### 2.7. Statistical Analysis

All statistical analyses were performed using STATISTICA software, version 13 (TIBCO Software Inc., Palo Alto, CA, USA). Prior to analysis, bacterial and phage counts were log_10_-transformed to meet the assumptions of normality. A Shapiro–Wilk test was performed to assess the normality of data. Differences in bacterial and phage concentrations in litter and feces samples over time (statistical unit: pen) were evaluated using analysis of variance (ANOVA), followed by the least significant difference (LSD) post hoc test for pairwise comparisons. Mortality data were analyzed using post hoc Tukey’s test. A *p*-value of <0.05 was considered statistically significant. All tests were two-tailed. Comparisons of bacterial load and phage load recovered from cecal contents of birds (statistical unit: individual bird) at the end of the experiment were performed using the nonparametric Mann–Whitney U test, due to the non-normal distribution of these data.

## 3. Results

### 3.1. Effect of UPWr_E Phage Cocktail on Total and Antimicrobial-Resistant Escherichia coli in Litter

**Figure 1:**
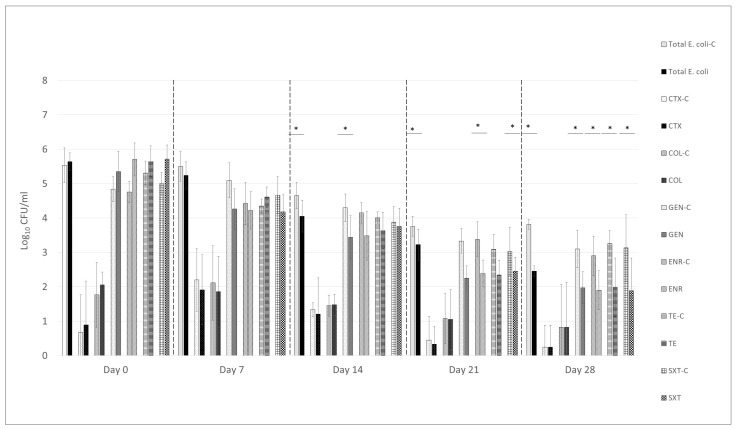
Proportions of total and resistant *E. coli* isolated from litter samples (*n* = 6 from 120 broilers per group) were recorded on study days 0, 7, 14, 21, and 28. Antimicrobials tested included CTX (cefotaxime), COL (colistin), GEN (gentamicin), ENR (enrofloxacin), TET (tetracycline), and SXT (sulfamethoxazole with trimethoprim). The “-c” designation indicates data from the untreated control group. * represents *p* < 0.05 and indicates a significant difference between groups.

Throughout the 4-week rearing period of broiler chickens, a gradual decline in the total *E. coli* was observed in the litter of the untreated (control) group, with a reduction of approximately 1.7 log_10_ CFU/g by the end of the experiment (Figure 1). In contrast, litter treated with a UPWr_E phage cocktail exhibited a significantly greater reduction in total *E. coli* counts, reaching a decrease of 3.2 log_10_ CFU/g relative to baseline levels, representing a 1.5 log_10_ CFU/g greater reduction compared to the control group (Figure 1; *p* < 0.05). A statistically significant decline (*p* < 0.05) in the number of *E. coli* isolates resistant to gentamicin, enrofloxacin, tetracycline, and the combination of sulfamethoxazole with trimethoprim was also observed in the phage-treated group. For gentamicin-resistant *E. coli*, the difference between the treated and control groups became apparent as early as two weeks after the initiation of phage application and remained statistically significant throughout the experiment. By the final day of the study, the reduction in *E. coli* resistant to gentamicin had reached 3.4 log_10_ CFU/g in the phage-treated group, compared to 1.9 log_10_ CFU/g in the control group. Similarly, for enrofloxacin-resistant *E. coli*, a significant decline was observed beginning three weeks post-treatment, with reductions of 3.8 log_10_ CFU/g and 1.9 log_10_ CFU/g in the phage-treated and control groups, respectively, by the end of the experiment (Figure 1). *E. coli* resistant to tetracycline exhibited reductions of 3.7 log_10_ CFU/g in the treated group versus 2.1 log_10_ CFU/g in the control group. A comparable trend was observed for isolates resistant to sulfamethoxazole combined with trimethoprim, with reductions of 3.8 log_10_ CFU/g in the phage-treated group and 1.9 log_10_ CFU/g in the untreated group. However, no significant differences were found between the treated and untreated groups in the number of *E. coli* growing in the presence of cefotaxime or colistin (Table S1; *p* > 0.05).

### 3.2. Effect of UPWr_E Phage Cocktail on Total and Antimicrobial-Resistant Escherichia coli in Feces

**Figure 2:**
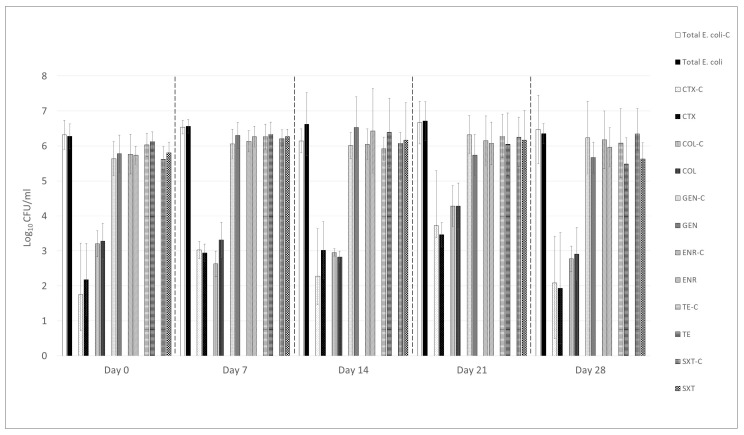
Proportions of resistant *E. coli* isolated from broilers’ feces (*n* = 6 from 120 broilers per group) were recorded on study days 0, 7, 14, 21, and 28. Antimicrobials tested included CTX (cefotaxime), COL (colistin), GEN (gentamicin), ENR (enrofloxacin), TET (tetracycline), and SXT (sulfamethoxazole with trimethoprim). The “-c” designation indicates data from the untreated control group.

Administration of the UPWr_E bacteriophage cocktail over 4 weeks did not result in a statistically significant reduction in the total number of *E. coli* present in the feces of treated chickens compared to untreated controls (Figure 2). Throughout the study, the average bacterial load of total *E. coli* remained within the range of 6.3 to 6.7 log_10_ CFU/g of feces in both the control and phage-treated groups, with no significant differences observed (*p* > 0.05). Furthermore, quantitative analysis of *E. coli* resistant to cefotaxime, colistin, gentamicin, enrofloxacin, tetracycline, and the combination of sulfamethoxazole with trimethoprim also showed no statistically significant differences between experimental groups (*p* > 0.05). Specifically, the abundance of cefotaxime-resistant *E. coli* ranged from 1.8 to 3.7 log_10_ CFU/g in the control group and from 1.9 to 3.5 log_10_ CFU/g in the phage-treated group. For colistin-resistant *E. coli*, bacterial counts varied from 2.6 to 4.3 log_10_ CFU/g in the control group and from 2.8 to 4.3 log_10_ CFU/g in the phage-treated group. Gentamicin-resistant *E. coli* were present in chicken feces at concentrations ranging between 5.6 and 6.3 log_10_ CFU/g in the control group and between 5.7 and 6.5 log_10_ CFU/g in the phage-treated group. Similarly, enrofloxacin-resistant *E. coli* counts ranged from 5.8 to 6.2 log_10_ CFU/g and from 5.7 to 6.4 log_10_ CFU/g in the control and treated groups, respectively. The bacterial load of tetracycline-resistant *E. coli* in feces was estimated to range from 5.9 to 6.3 log_10_ CFU/g in the control group and from 5.5 to 6.4 log_10_ CFU/g in the phage-treated group. Lastly, the enumeration of *E. coli* resistant to sulfamethoxazole combined with trimethoprim revealed similar levels in both groups, with values ranging from 5.6 to 6.3 log_10_ CFU/g. No differences were observed between pens within the same experimental group.

### 3.3. Evaluation of the Impact of the UPWr_E Bacteriophage Cocktail on Total and Antimicrobial-Resistant Escherichia coli in the Cecal Contents of Broiler Chickens

**Figure 3:**
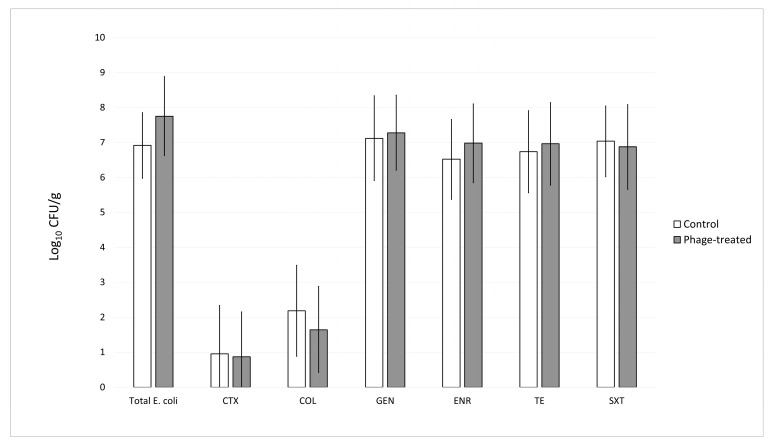
Effect of phage cocktail UPWr_E on total and antibiotic-resistant *E. coli* levels in chicken cecal contents. Bacterial loads are shown as counts for individual birds (*n* = 18 per group) of the control group (white bars) and the phage-treated group (grey bars). Antimicrobials tested included CTX (cefotaxime), COL (colistin), GEN (gentamicin), ENR (enrofloxacin), TET (tetracycline), and SXT (sulfamethoxazole combined with trimethoprim).

Quantitative bacteriological analysis revealed that phage treatment did not result in a statistically significant reduction in the total *E. coli* population in the cecal contents of treated animals compared to the control group (Figure 3). Throughout the experimental period, the bacterial load of total *E. coli* in the cecal samples from birds from the control group ranged from 5.3 to 8.1 log_10_ CFU/g, whereas in the phage-treated group, values ranged from 5.8 to 9.4 log_10_ CFU/g. No significant differences were observed between groups at any time point (*p* > 0.05). In addition to total *E. coli* counts, the prevalence and abundance of antibiotic-resistant *E. coli* strains were assessed using selective culturing methods targeting resistance to cefotaxime, colistin, gentamicin, enrofloxacin, tetracycline, and the combination of sulfamethoxazole with trimethoprim. Across all antimicrobial categories, the application of the UPWr_E phage cocktail did not significantly affect the number of resistant *E. coli* isolates when compared with untreated controls (*p* > 0.05). Specifically, *E. coli* strains resistant to cefotaxime were detected in 6 out of 18 chicks in the control group, with counts ranging from 2.4 to 3.0 log_10_ CFU/g, and in 5 birds from the phage-treated group, where values ranged from 2.0 to 3.0 log_10_ CFU/g. For colistin-resistant *E. coli*, bacterial concentrations ranged from 2.0 to 3.6 log_10_ CFU/g in the control group, with four birds testing negative, and from 2.1 to 2.9 log_10_ CFU/g in the phage-treated group, with 5 birds yielding negative results. Gentamicin-resistant *E. coli* were identified at levels between 5.3 and 8.8 log_10_ CFU/g in cecal contents of birds from the control group and between 5.7 and 9.5 log_10_ CFU/g in the phage-treated group. Similarly, enrofloxacin-resistant *E. coli* were detected in the ranges of 5.3 to 9.2 log_10_ CFU/g and 5.6 to 9.1 log_10_ CFU/g in the control and phage-treated groups, respectively. The bacterial load of tetracycline-resistant *E. coli* ranged from 5.0 to 9.5 log_10_ CFU/g in samples of cecal contents from the control group and from 5.3 to 9.2 log_10_ CFU/g in samples from the phage-treated group. Finally, the enumeration of *E. coli* resistant to sulfamethoxazole combined with trimethoprim revealed comparable results between groups, with concentrations ranging from 5.3 to 8.1 log_10_ CFU/g in the control group and 5.8 to 8.9 log_10_ CFU/g in the phage-treated group. No differences were observed between pens within the same experimental group.

### 3.4. Phage Recovery from Litter, Feces and Cecal Samples

**Figure 4:**
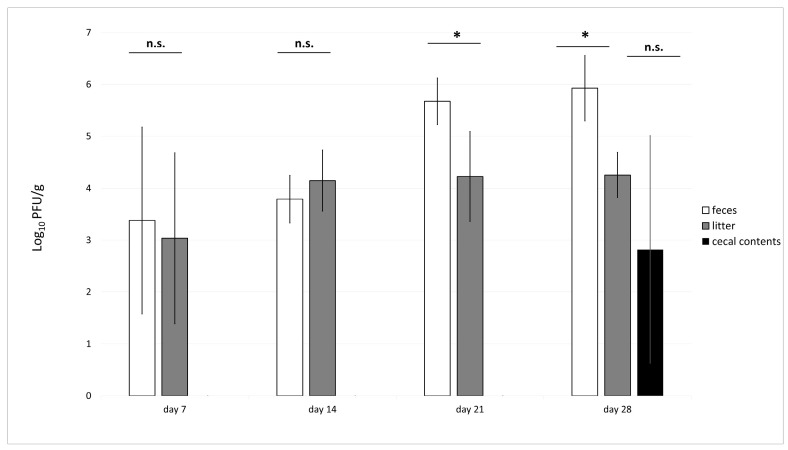
UPWr_E phage titers in broiler feces, litter, and cecum. Results for phage load are shown as counts for individual animals (*n* = 18 per group) for the phage-treated group in feces (white bars), litter (grey bars), recorded on study days 0, 7, 14, 21, and 28 and in cecal contents (black bar) on day 28. * represents *p* < 0.05 and indicates a significant difference between groups; n.s., not significant.

One week following the initial bacteriophage administration, phages were detected in litter samples collected from five out of six monitored pens, while one pen remained phage-negative. The phage titers in these positive samples ranged between 2.7 and 4.6 log_10_ PFU/g (Figure 4). Following the second phage treatment, phage titer was estimated to vary between 3.3 and 4.6 log_10_ PFU/g. After 3 weeks of phage treatment, the detected phage concentrations in the litter increased, with titers ranging from 3.7 to 5.9 log_10_ PFU/g. Subsequent administrations maintained detectable phage levels, with titers ranging from 3.8 to 4.8 log_10_ PFU/g. A similar trend was observed in fecal samples. One week after the initial phage treatment, phages were not detected in feces from one of the pens, whereas the remaining samples tested positive, with phage titers ranging between 3.5 and 4.9 log_10_ PFU/g. Two weeks post-treatment, phages were detected in all samples, with fecal phage concentrations ranging from 3.2 to 4.8 log_10_ PFU/g, and by the third week, an increase in titer was observed, with values ranging between 4.8 and 5.9 log_10_ PFU/g. On the final day of the experiment, phage titers in the litter remained within a range of 3.7 to 4.8 log_10_ PFU/g. In fecal samples, the phage concentration after one week of treatment continued to fall within the previously observed range of 3.5 to 4.9 log_10_ PFU/g among the five phage-positive samples. Following the second administration, the phage titers ranged from 3.3 to 4.6 log_10_ PFU/g. A subsequent treatment further elevated the concentration to 4.8–5.9 log_10_ PFU/g. Ultimately, after a total of four phage applications, the phage load in feces reached its highest level, ranging from 5.5 to 6.8 log_10_ PFU/g. Throughout the experimental timeline, the phage concentration consistently remained lower in litter samples compared to fecal samples. Cecal contents analysis revealed 12 positive samples out of 18 containing phages at titers between 2.2 and 5.3 log_10_ PFU/g.

### 3.5. Growth Performance Parameters

The impact of litter treatment with UPWr_E phage cocktail on growth performance parameters, including body weight, feed intake, and feed conversion ratio, is presented in Table 1. The results demonstrated that phage treatment had no statistically significant effect on feed intake, final body weight, or mortality.

## 4. Discussion

This study investigated the effect of the UPWr_E bacteriophage cocktail on both total and antimicrobial-resistant *E. coli* in litter, feces, and cecal content. The pronounced reduction in *E. coli* in the litter, particularly those strains resistant to gentamicin, enrofloxacin, tetracycline, and sulfamethoxazole combined with trimethoprim, highlights the efficacy of the UPWr_E phage cocktail in mitigating environmental reservoirs of antimicrobial-resistant bacteria. A 3.2 log_10_ CFU/g reduction in total *E. coli* and a notably greater reduction in resistant strains, compared to controls, indicate that the phages retained lytic activity under on-farm conditions. Poultry litter, a major by-product of broiler meat production, is commonly used as a nutrient-rich fertilizer and is frequently applied as a soil amendment [20]. However, application of animal-derived manures, including poultry litter, poses potential public health risks due to the potential presence of zoonotic pathogens [21,22,23]. Gram-negative bacteria, including foodborne pathogens such as pathogenic *E. coli*, *Campylobacter* sp., and *Salmonella* spp., constitute a relatively minor fraction of the overall poultry litter microbiota [20]. However, the commensal *E. coli* population usually reaches 10^6^ CFU/g of intestinal content [24]. *E. coli* strains originating from poultry frequently display multidrug resistance, commonly associated with mobile genetic elements that can capture and disseminate antimicrobial resistance gene cassettes [25,26,27]. It was clearly shown that numerous AMR genes are shared among disparate bacterial members of the litter microbiota [28]. Our findings demonstrate that phage application significantly reduced both total *E. coli* and *E. coli* resistant to gentamicin, enrofloxacin, tetracycline, and sulfamethoxazole combined with trimethoprim in the litter, while exerting no detectable impact on the intestinal microbial load or resistance profiles within the gastrointestinal tract. Therefore, a reduced burden of resistant *E. coli* in litter after the rearing period could substantially limit the environmental dissemination of AMR genes to soil, water, and crops. These findings support the potential role of phage-based interventions as part of sustainable on-farm AMR control strategies.

The administration of bacteriophages, either directly to poultry or via aerosolized delivery, has been extensively reviewed for its therapeutic potential in controlling bacterial infections [17]. El-Gohary et al. (2014) [14] reported that surface application of a bacteriophage preparation at a titer of 8 × 10^8^ PFU/mL significantly reduced mortality in male broiler chickens suffering from colibacillosis due to APEC exposure in the litter. Notably, the protective effect was maintained even under cold stress conditions and was accompanied by a reduction in pathogen shedding within the flock. Recently, Lopes et al. (2025) [13] observed that treatment with the VAM-S bacteriophage significantly influenced the microbial composition and diversity of the poultry litter microbiota. The findings underscore the potential of VAM-S bacteriophage treatment to beneficially modulate the poultry litter microbiome by reducing pathogen load and promoting the growth of beneficial bacterial populations. According to the authors, litter treatment with bacteriophages presents a promising alternative to antibiotics, particularly in light of growing consumer concerns regarding antimicrobial use in animal production. Bacteriophages offer a sustainable strategy for enhancing food safety, with a lower propensity for resistance development in comparison to conventional antimicrobials [29]. While the use of bacteriophages as targeted biocontrol agents against pathogenic and undesirable bacteria in poultry has been the focus of extensive research and is reviewed elsewhere [30,31], there remains a significant gap in knowledge regarding their potential application as effective tools for mitigating AMR within animal production systems. Despite growing interest in phage therapy and its theoretical advantages, such as high specificity, self-amplification at the site of infection, and minimal impact on the commensal microbiota, empirical evidence supporting its efficacy in reducing AMR reservoirs in livestock environments is currently limited. Our study clearly showed the high effectiveness of bacteriophage-based interventions to selectively target and reduce populations of antimicrobial-resistant bacteria, as well as to assess their integration into broader antimicrobial stewardship strategies in agriculture.

Despite this environmental efficacy, the lack of significant reduction in both total and resistant *E. coli* in feces and cecal content suggests a neutral mode of action towards the microbiota. One possible explanation is the limited penetration or activity of the phages within the gastrointestinal tract due to the complexity and density of the gut microbiota, which could hinder phage access to target bacteria or lead to rapid phage inactivation. Kittler et al. (2020) [32] reported that administration of a four-phage cocktail at a concentration of 4.6 log_10_ PFU/mL in drinking water resulted in a reduction in *E. coli* counts in chicken feces. In contrast, treatment with a six-phage cocktail at a higher concentration of 6.7 log_10_ PFU/mL did not produce a significant effect. The authors suggested that the dosage of the phage cocktail plays a critical role in determining its efficacy against *E. coli*. However, as highlighted previously, the multiplicity of infection (MOI) based on adsorption, defined as the number of phages that adsorb to each bacterium, is a more accurate indicator of therapeutic effectiveness [33]. This parameter depends on both the mass and concentration of adsorbing particles at the infection site and may have been higher in the six-phage treatment group due to an increased bacterial density [33,34]. In our research, the neutral effect on the number of commensal *E. coli* observed in the cecal microbiota may suggest that the absence of *E. coli* reduction did not substantially influence the composition of the remaining microbiota. In broilers, commensal *E. coli* typically represents a very small fraction of the cecal microbiota estimated to be approximately 0.1%, making it unlikely that an unchanged *E. coli* count would drive community-wide shifts [35]. The results provide a suggestion that the UPWr_E phage cocktail did not interfere with the natural microbial balance. The above findings collectively indicate that the administration of the UPWr_E phage cocktail on litter, under the experimental conditions employed, did not produce measurable effects on the total *E. coli* population or on the prevalence of antimicrobial-resistant *E. coli* strains within the gastrointestinal tract of broiler chickens. These findings are consistent with the lack of statistically significant effects of the tested intervention on feed intake or final body weight in chickens, thereby supporting the notion that the presence of phages does not markedly influence these growth performance parameters.

Interestingly, the absence of a detectable phage effect on *E. coli* resistant to cefotaxime and colistin across all sampled environments warrants further investigation. One possible explanation is the markedly lower baseline prevalence of these resistant *E. coli*, which may have reduced the likelihood of phage-host encounters. Additionally, these clones might lack the specific receptors targeted by the phage cocktail or occupy ecological niches that are less accessible to phage particles. This observation highlights the importance of accounting for both bacterial abundance and host range specificity in phage therapy design. It also suggests that phage cocktails intended for broad-spectrum control of antimicrobial resistance may require periodic reformulation or supplementation to remain effective against shifting resistance profiles.

The successful recovery of active phages from both litter and feces throughout the treatment period confirms their environmental stability and persistence. The phage burden within the litter remained relatively stable throughout the experiment, confirming our previous findings on phage stability and efficacy under various conditions [16,36]. The phage concentration consistently remained lower in litter samples compared to fecal samples, indicating differential persistence or accumulation of phages depending on the sample matrix, likely due to direct passage through the gastrointestinal tract. Moreover, detectable phage presence in cecal contents demonstrates that presence of phages in the litter can lead to phage delivery to the distal gut, even if not at uniformly high titers or with an observable antimicrobial effect in this compartment.

This study has several limitations that should be acknowledged. First, *E. coli* enumeration was performed using MacConkey agar, which primarily supports the growth of members of the *Enterobacteriaceae* family. Although lactose-fermenting *E. coli*-like colonies were selected based on morphology, other species may also have grown on the medium. All isolation procedures followed EUCAST/CLSI guidelines for non-specific *E. coli* recovery. Second, while phages were detected in the gut, their stability, replication dynamics, and spatial distribution within the intestinal environment remain unclear, limiting our understanding of their in vivo activity. Third, the study did not assess the potential emergence of phage-resistant bacterial mutants, nor did it employ large sample sizes or long-term monitoring strategies. To address these gaps and enhance the effectiveness of phage-based interventions in poultry farming, future research should incorporate metagenomic approaches, expanded cohort sizes, and longitudinal tracking of resistance evolution.

## 5. Conclusions

This study demonstrated that the UPWr_E bacteriophage cocktail effectively reduced both total and antimicrobial-resistant *E. coli* populations in poultry litter, while having no observable impact on the gastrointestinal number of commensal *E. coli* in cecal contents of broiler chickens. The marked reduction in *E. coli* resistant to gentamicin, enrofloxacin, tetracycline and a combination of sulfamethoxazole with trimethoprim in litter highlights the potential of phage therapy to mitigate environmental reservoirs of AMR in agricultural settings. Despite limited efficacy within the gut, the environmental stability and persistence of phages support their feasibility as a non-disruptive, targeted intervention. Overall, these findings support the integration of bacteriophage-based strategies into sustainable on-farm AMR control programs to enhance food safety and environmental health.

## Figures and Tables

**Table 1:** Growth performance parameters (means) of broiler chickens during the 4-week rearing period.

Parameter	Control	Phage-Treated	SEM	*p* Value
Body weight (g)	2490	2606	71.52	0.444
Feed intake (g)	3592	3720	75.06	0.421
Feed conversion ratio	1.445	1.431	0.012	0.586
Mortality (%)	8.333	4.170	-	0.103
European Production Efficiency Factor	377	417	15.12	0.201

## Data Availability

The original contributions presented in the study are included in the article; further inquiries can be directed to the corresponding author.

## Supplementary Materials

The following supporting information can be downloaded at: https://www.mdpi.com/article/10.3390/ani15172525/s1, Table S1. Log differences in *E. coli* loads between control and phage-treated group in litter, feces, and cecal contents. Negative values indicate cases where the highest *E. coli* counts in the phage-treated group exceeded those in the control group.

## Abbreviations

The following abbreviations are used in this manuscript:

AMR	Antimicrobial resistance
CFU	Colony forming unit
APEC	Avian pathogenic *Escherichia coli*
EFSA	European Food Safety Authority
OD	Optical density
PFU	Plaque forming units
PBS	Phosphate-buffer saline
EUCAST	European Committee on Antimicrobial Susceptibility Testing
CLSI	Clinical and Laboratory Standards Institute
FCR	Feed conversion ratio
MOI	Multiplicity of infection
