# Supplementary material for: Environmental Application of a Bacteriophage Cocktail Reduces Antibiotic-Resistant Escherichia coli in Poultry Litter Without Disrupting Gut Microbiota
Source: Animals (Basel). 2025 Aug 27;15(17):2525. doi: 10.3390/ani15172525 (PMC12427302; doi:10.3390/ani15172525)
Supplement: Supplementary file 1 [file animals-15-02525-s001.zip › animals-3801636-supplementary.pdf]

Table S1. Log differences in *E. coli* loads between control and phage-treated group in litter, feces, and cecal contents. Negative values indicate cases where the highest *E. coli* counts in the phage-treated group exceeded those in the control group.

**Litter**							
	Total *E. coli*	CTX	COL	GEN	ENR	TE	SXT
Day 0	-0,10078	-0,21684	-0,28865	-0,51061	-0,95442	-0,33461	-0,71238
Day 7	0,267926	0,285201	0,255125	0,833484	0,204227	-0,26234	0,481597
Day 14	0,601036	0,121166	-0,02963	0,855783	0,671338	0,362682	0,131882
Day 21	0,531981	0,116495	0,032716	1,080419	0,995329	0,753813	0,575989
Day 28	1,361189	0	0	1,128793	0,997384	1,260905	1,243571
**Feces**							
	Total *E. coli*	CTX	COL	GEN	ENR	TE	SXT
Day 0	0,040506	-0,42803	-0,07377	-0,14085	0,032303	-0,10034	-0,19102
Day 7	-0,02932	0,084778	-0,68302	-0,24327	-0,13562	-0,07024	-0,06448
Day 14	-0,477	-0,74016	0,124883	-0,51476	-0,37801	-0,46386	-0,09384
Day 21	-0,04008	0,26701	0,000909	0,575426	0,080469	0,231291	0,083451
Day 28	0,121486	0,150515	-0,13732	0,570049	0,214234	0,596298	0,722091
**Cecal content**							
	Total *E. coli*	CTX	COL	GEN	ENR	TE	SXT
Day 28	-0,83597	0,080398	0,537414	-0,15741	-0,45982	-0,22706	0,161751
